# Molecular Recognition by a Polymorphic Cell Surface Receptor Governs Cooperative Behaviors in Bacteria

**DOI:** 10.1371/journal.pgen.1003891

**Published:** 2013-11-07

**Authors:** Darshankumar T. Pathak, Xueming Wei, Arup Dey, Daniel Wall

**Affiliations:** Department of Molecular Biology, University of Wyoming, Laramie, Wyoming, United States of America; University of California, Santa Barbara, United States of America

## Abstract

Cell-cell recognition is a fundamental process that allows cells to coordinate multicellular behaviors. Some microbes, such as myxobacteria, build multicellular fruiting bodies from free-living cells. However, how bacterial cells recognize each other by contact is poorly understood. Here we show that myxobacteria engage in recognition through interactions between TraA cell surface receptors, which leads to the fusion and exchange of outer membrane (OM) components. OM exchange is shown to be selective among 17 environmental isolates, as exchange partners parsed into five major recognition groups. TraA is the determinant of molecular specificity because: (i) exchange partners correlated with sequence conservation within its polymorphic PA14-like domain and (ii) *traA* allele replacements predictably changed partner specificity. Swapping *traA* alleles also reprogrammed social interactions among strains, including the regulation of motility and conferred immunity from inter-strain killing. We suggest that TraA helps guide the transition of single cells into a coherent bacterial community, by a proposed mechanism that is analogous to mitochondrial fusion and fission cycling that mixes contents to establish a homogenous population. In evolutionary terms, *traA* functions as a rare greenbeard gene that recognizes others that bear the same allele to confer beneficial treatment.

## Author Summary

How individual cells recognize each other to cooperate and assemble functional tissues is a fundamental question in biology. Although multicellularity is a trait that is typically associated with eukaryotes, certain groups of bacteria also exhibit complex multicellular behaviors, which are perhaps best exemplified by the myxobacteria. For example, in response to starvation myxobacteria will assemble fruiting bodies, wherein thousands of cells function as a coherent unit in development and cell differentiation. However, how myxobacteria, or for that matter other bacteria, recognize cooperating partnering cells through cell contact-dependent interactions is poorly understood. Here we describe a mechanism where myxobacteria distinguish sibling and cohort cells from other myxobacteria isolates. We show that molecular recognition is mediated by a cell surface receptor called TraA. Cell-cell specificity involves mutual recognition by partnering cells and is mediated by proposed homotypic TraA interactions. The specificity for recognition is determined by variable sequences found within *traA* alleles. Thus, simply swapping *traA* alleles between isolates predictably changes partner recognition. TraA-TraA recognition in turn leads to the fusion and exchange of outer membrane (OM) components between cells. We suggest that OM exchange allows the cells to communicate and become homogenous with respect to their OM proteome. We further suggest these interactions build a cohesive cell population that functions in multicellular processes.

## Introduction

Cell-cell recognition is critical for differentiating friend from foe and for allowing populations of cells to coordinate multicellular functions [1], [2]. Many eukaryotes simplify aspects of cellular self-recognition by clonal expansion from a single fertilized cell, wherein a privileged environment excludes nonself cells. In contrast, some eukaryotes and bacteria build multicellular structures from heterogeneous free-living cells in the environment. In these cases, coalescing cells are not necessarily siblings or even the same species [1], [3], [4]. Therefore, mechanisms involved in cell-cell recognition are required to ensure selective inclusion of cells into cooperative multicellular cohorts. In the case of bacteria, however, little is known about how cells physically recognize one another to coordinate multicellular functions.

Myxobacteria represent an attractive model system to understand bacterial cell-cell recognition, because they have complex social behaviors in which cells are recruited from their environment to perform multicellular tasks. For instance, during vegetative growth, myxobacteria can exist as solitary cells or as small groups of cells; upon starvation they transition into large, organized multicellular cohorts that build erect macroscopic fruiting bodies [5]. The ability of myxobacteria to cobble together a coherent population of cells from environments rich in microbial diversity [6] implies that they have a mechanism(s) to identify and sort closely related cells from distantly related cells. To date, however, no molecular recognition system has been characterized in myxobacteria. Recently, we discovered a novel social interaction in myxobacteria that suggests a role for cell discrimination. This behavior involves the mutual exchange of outer membrane (OM) lipids and proteins between cells [7]–[9]. In contrast, no cytoplasmic or DNA material is exchanged. The output of these interactions includes phenotypic changes to cells and provides a conduit for cell-cell communication [5]. Strikingly, OM exchange involves sharing of large quantities of OM material, i.e. of the components that are transferred are essentially equally divided between interacting cells [7]–[9]. We therefore hypothesized that myxobacteria might have evolved a mechanism to discriminate among candidate partner cells before they commit to the energetically costly behavior of sharing large quantities of cellular material.

Insight into the mechanism of OM exchange was made with the identification of the TraA and TraB proteins [7]. With the use of fluorescent reporters, TraAB were shown to be required in both ‘donor’ and ‘recipient’ cells for transfer. Thus, unlike known bacterial secretion or conjugation systems, in which one cell expresses a transport machine to unidirectionally deliver cargo to target cells, the TraAB system instead requires that both cells express the transfer machinery. In other anthropomorphic words, the decision to exchange material is mutually made by interacting cells because both cells must functionally express TraAB. Fluorescently labeled lipids also transfer in a TraAB-dependent manner [7], and thus the OMs apparently transiently fuse and cargo diffuses or exchanges bidirectionally between cells. TraAB are predicted to reside in the cell envelope, and, because TraAB overexpression results in cells that adhere together in chains, TraA may function as a cell surface adhesin and thus could play a role in cell recognition [7].

The exchange of OM proteins results in phenotypic and behavioral changes in those cells. For instance, certain gliding motility mutants are rescued or complemented extracellularly by protein transfer from a ‘donor’ strain that expresses the corresponding wild-type protein [10]. In the case of *tgl* mutants, which are defective in assembling their motor, type IV pili, physical contact with a *tgl* ^+^ cell results in transfer of the Tgl lipoprotein to the mutant ‘recipient’ [9], [11], [12]. Once Tgl function is provided, the mutant assembles its type IV pili and the cell therefore can move. Because no DNA is exchanged, the phenotypic rescue of motility is transient, as the Tgl protein is diluted over time by protein turnover and cell divisions. In other examples, OM exchange serves as a conduit for cell-cell signaling [5]. In such cases, one strain can regulate the behavior of another strain with regard to the decision to expand the swarm or to enter fruiting body development.

In this work, we sought to address the question of whether myxobacteria use cell recognition to identify partnering cells for OM exchange. By using a panel of environmental isolates, we show that OM exchange is selective and that TraA is a polymorphic receptor that determines specificity.

## Results

### Myxobacteria form discrete recognition groups to conduct OM exchange

**Figure 1:**
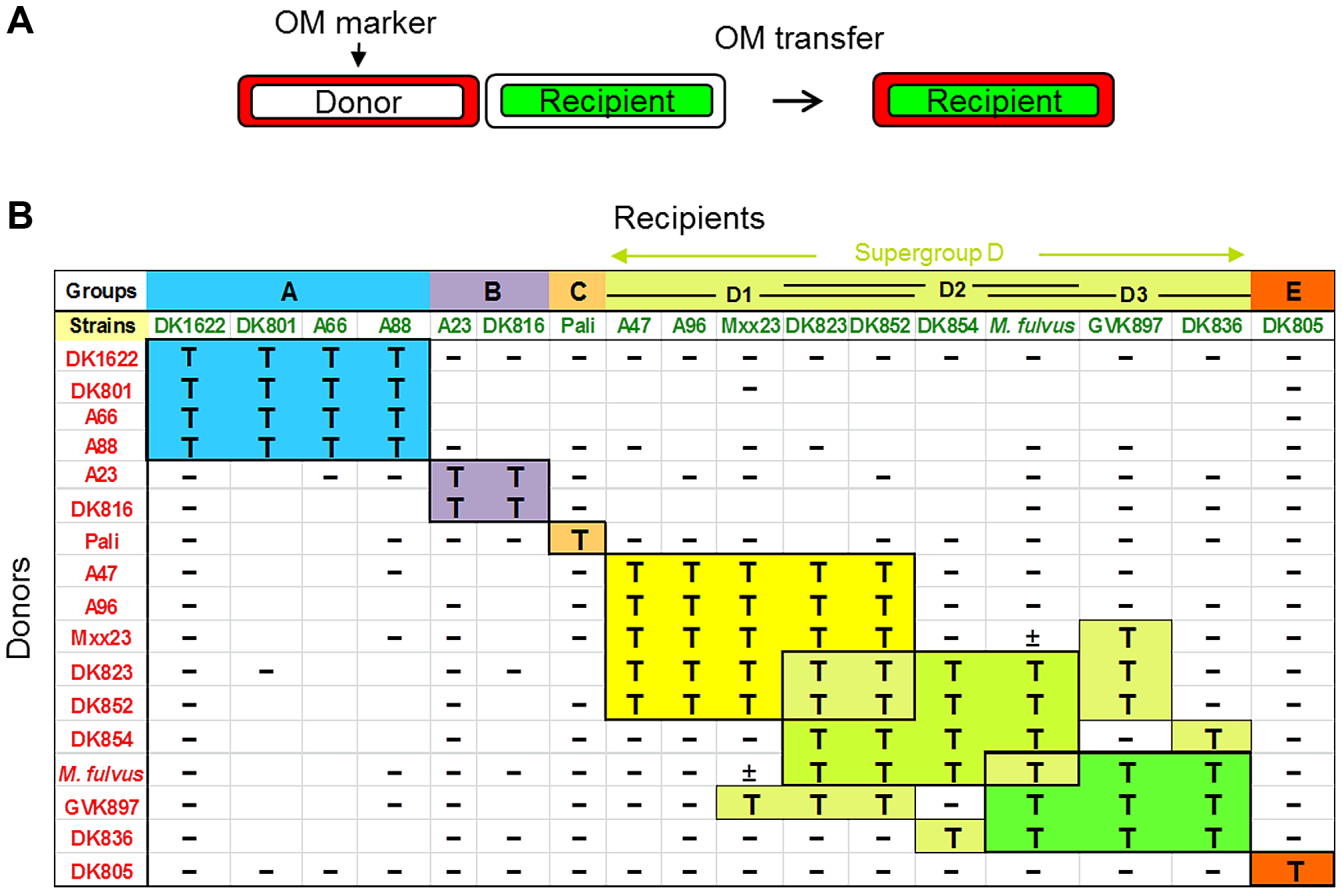
OM exchange is strain specific. A) Schematic for how OM exchange was scored. Donor cell OMs were labeled with either lipophilic or lipo (SS_OM_)-mCherry red fluorescent reporters. Transfer was determined by the ability of labeled green fluorescent recipient cells to turn red. B) Assessment of 16 independent *M. xanthus* isolates and a closely related *M. fulvus* species for their ability to exchange OM components. Distinct recognition groups are color coded. Donors and recipients indicate the direction of transfer. T, transfer; minus (−), no transfer; ±, poor transfer. Strain mixtures that were not tested are indicated as blank boxes.

The finding that myxobacteria engage in intimate cellular resource sharing led us to hypothesize that this process may involve a form of self/nonself recognition. To address this possibility, we mixed a double-labeled laboratory strain containing cytoplasmic green fluorescent protein (GFP), which does not transfer, and a transferable OM mCherry lipoprotein reporter (SS_OM_-mCherry) with individual strains from a panel of unlabeled environmental *Myxococcus xanthus* isolates (Table S1) [8]. The laboratory strain (a DK1622 derivative) transferred SS_OM_-mCherry to only 3 of 15 isolates (data not shown). These results suggested that OM exchange was indeed selective; however, some strains might not be transfer competent. To address this possibility, we developed an analogous transfer assay in which donor cell membranes were stained with a lipophilic red dye (membrane transfer) and recipient cells were stained with a cytoplasmically trapped green fluorescent conjugate that cannot transfer (Fig. 1A). This assay again requires TraAB function for lipophilic red fluorescent dye transfer [7]. In this new assay, the 12 isolates that could not transfer with the lab strain were indeed found to be competent for transfer with themselves (Fig. 1B). Thus the failure of the laboratory strain to transfer with certain isolates was due to selectivity, not functional competence.

Next, we carried out comprehensive tests for inter-strain transfer among 17 isolates, which included the addition of a closely related *M. fulvus* species. In total 213 transfer combinations were tested. Interestingly, these experiments found that OM exchange was restricted to particular partners, which are henceforth called recognition groups (Fig. 1B). For example, group A contains four members that all transferred amongst themselves, but not with other isolates. Similarly, group B contains two members that transferred only between themselves. In the cases of the Pali and DK805 isolates, they were highly selective and transferred only to themselves and thus represent single-member groups (C and E, respectively). The remaining strains were classified into a large, loosely defined supergroup designated D. Unlike other groups, transfer among D members was somewhat heterogeneous and was divided into subgroups (D1, D2 and D3) that exchanged among themselves. Unlike other recognition groups, promiscuous transfer did, however, occur between some subgroup D members. For instance, DK823 and DK852 transferred with all supergroup D members except DK836.

Since our assays are designed to detect only unidirectional transfer between two strains, we carried out reciprocal experiments by reversing the fluorescent labels each strain was stained with, to test for bidirectional transfer. In every case tested, 94 pairs of different strains (188 assay combinations), the reciprocal transfer experiment gave the identical result (Fig. 1B).For example, DK823 transferred to A96 and A96 transferred to DK823, while in contrast DK823 did not transfer to DK1622 and DK1622 did not transfer to DK823 (Fig. 1B). These results therefore support the idea that transfer is selective and bidirectional [7].

### TraA is polymorphic within the PA14-like domain

**Figure 2:**
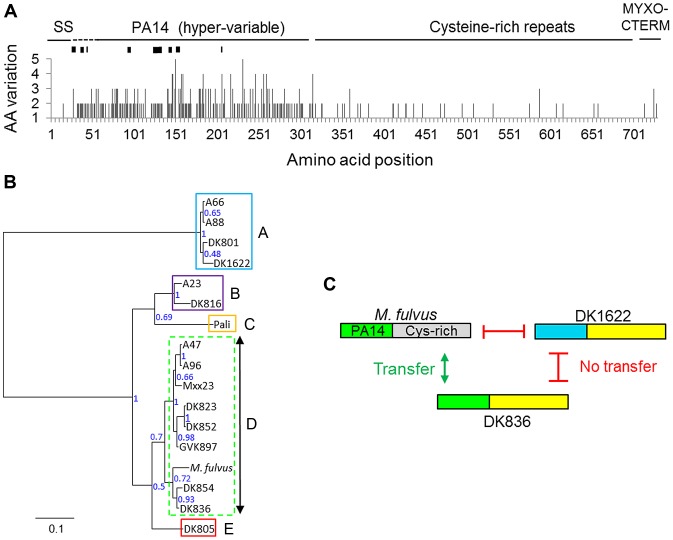
The TraA PA14-like domain is polymorphic and correlates to recognition groupings. A) TraA amino acid (AA) variation derived from a sequence alignment from 16 *M. xanthus* strains is plotted. *M. fulvus* represents a distinct species and was excluded. Black rectangles in the hyper-variable region represent indels that range from one to seven codons in length. The signal sequence (SS) and a putative protein sorting tag (MYXO-CTERM) are also labeled. B) Phylogenetic tree derived from the PA14 polymorphic region, unrooted. Node support values are given as posterior probabilities. The multiple-sequence alignment used to generate the tree is provided in Figure S1. Recognition groups are boxed and labeled. A dashed border indicates the heterogeneous recognition group. C) Domain similarity between three TraA sequences is graphically depicted and color coded. Gray and blue regions contain divergent sequences. Transfer compatibility of TraA variants is shown by green arrows (transfer) or red bars (no transfer). Specificity was determined by PA14 domain relatedness. The apparent chimeric domain architecture, depicting sequence relatedness, suggests that DNA rearrangements occurred between ancestral *traA* alleles. See Figure S2 for alignments.

The domain architecture of TraA consists of a type I signal sequence, a distant PA14-like domain, a cysteine-rich tandem repeat region, and a MYXO-CTERM motif postulated to function in protein sorting to the cell surface [7]. This domain architecture is similar to the FLO adhesin proteins found in yeast [13] and suggests that TraA may function as a cell surface receptor. To investigate whether TraA functions in cell-cell recognition, we sequenced the *traA* alleles from the environmental isolates and analyzed the sequences for possible polymorphisms. A sequence alignment was generated, and variable amino acid residues were plotted along the length of the protein (Fig. 2A). A hyper-variable region, which contains amino acid polymorphisms and indels, was found to encompass the PA14-like domain (Figs. 2A and S1). In contrast, the other regions in the protein showed little sequence variation (Fig. 2A). Therefore, the PA14-like domain is polymorphic, suggesting it may play a role in partner selectivity.

### TraA sequence polymorphisms correlate with recognition groupings

To test whether the described TraA sequence variations identified in Figure 2A might predict strain specificity, those sequences were compared with the described recognition groups (Fig. 1B). In our initial analysis, a correlation was suggested, because recognition groups A and D1 each contained two members with identical TraA sequences, A66/A88 and A47/A96, respectively. Additionally, DK823 and DK852 have only two amino acid differences and were D1 partners. We thus constructed a phylogenetic tree based on the variable region that encompasses the PA14-like domain to carry out a more comprehensive analysis. Importantly, this tree shows a strong correlation between *traA* genetic relatedness and recognition groupings—the recognition groups clustered almost perfectly according to the sequence conservation found in their PA14 domains (Fig. 2B). Based on amino acid substitutions and indels, group A is phylogenetically distant and constitutes an outgroup. Similarly, B, C and E also form distinct recognition groups that correlate to phylogenetic groupings (Fig. 2B). Supergroup D forms a clade that is more heterogeneous in terms of sequences and transfer partner recognition (Figs. 1B and 2B). Although there is some heterogeneity in supergroup D, there is nevertheless nearly perfect agreement between sequence conservation and recognition group partnering. These results indicate that the TraA protein sequence determines specificity among recognition groups.

An extension from the above finding suggests that the PA14-like domain could be functionally responsible for recognition. To examine this idea in more detail, we compared the full-length TraA protein sequence from *M. fulvus* with other *M. xanthus* sequences, because the former sequence has a divergent C-terminal sequence that encompasses the Cys-rich repeats (Figs. 2A and S2). The alignment of the *M. fulvus* sequence to the fellow recognition group D member DK836 and a representative from another recognition group (DK1622) showed striking results (Fig. S2). Although the C-terminal regions in TraA^DK1622^ and TraA^DK836^ were nearly identical over a >400-amino-acid region, they were not OM exchange partners (Figs. 2C and S2). In contrast, TraA^DK836^, which has a divergent C-terminal sequence from TraA*^M. fulvus^* but has a similar PA14-like domain, constitute transfer partners (Figs. 2C and S2). These findings support the idea that the PA14-like domain within TraA serves as the molecular recognition determinant.

### TraA localizes to the cell surface

**Figure 3:**
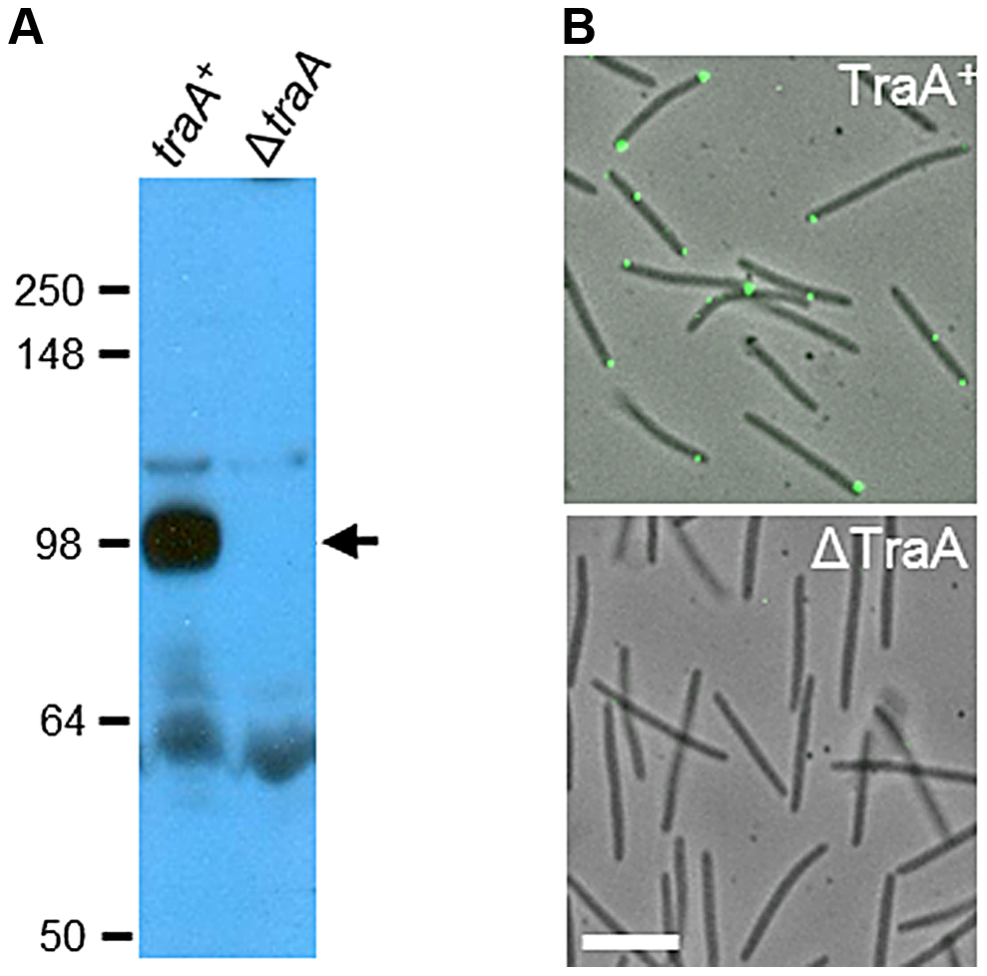
TraA is a cell surface receptor. A) Western blot with TraA-PA14 antibodies against whole-cell lysates from *traA* ^+^ (DW1463) and Δ*traA* (DW1467) strains. Molecular weight markers (kDa) are shown at the left, and the arrow indicates the TraA-specific band at ∼100 kDa. B) TraA immunofluorescence micrographs of live non-permeabilized cells. The same strains and primary antibodies were used as in A. White bar represents 2 µm.

To address the hypothesis that TraA functions as a cell surface receptor, polyclonal antibodies were raised against the PA14^DK1622^ domain. Whole-cell western blot analysis identified a single prominent ∼100-kDa band that was absent from a Δ*traA* strain (Fig. 3A). The migration of the TraA-specific band was slower than that of the calculated molecular weight of the full-length processed protein (71 kDa) and supports an earlier suggestion that TraA and particularly the MYXO-CTERM domain could be post-translationally modified [7]. Immunofluorescence microscopy was then conducted and found that TraA was detected on live non-permeabilized cells, whereas a Δ*traA* strain did not cross-react with the antibodies (Fig. 3B). These results indicate that the PA14-like domain of TraA localizes to the cell surface. The localization of TraA was further found to be enriched at cell poles (72% of the time; 321/445 of fluorescent foci counted). Large, bright foci were observed on some cells, perhaps suggesting that TraA may form receptor clusters (Fig. 3B). These findings support our earlier claims that transfer involves end-to-end cell contacts and is mediated by the TraA cell surface adhesin [7], [8], [14].

### TraA is the molecular specificity determinant

**Figure 4:**
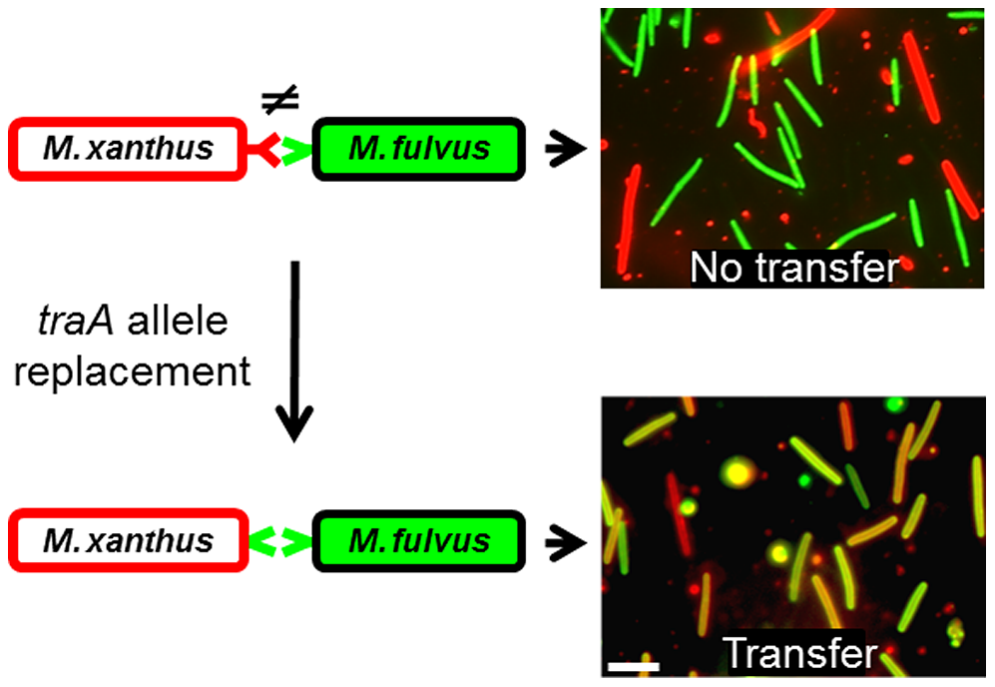
TraA is the molecular determinant for specificity. Schematic representations of cell-cell interactions are shown on the left, in which variant TraA receptors are color coded. On the right are merged micrographs from red and green fluorescence images after mixed cells were collected from an agar surface. The laboratory *M. xanthus* strain was labeled with a red lipophilic DiD membrane dye, which does not transfer to the *M. fulvus* cells, which were labeled with the green fluorescent tracer dye. In contrast, an isogenic *M. xanthus traA* allele replacement strain (DW1470), which encodes the *traA^M. fulvus^* allele, enables recognition and transfer with *M. fulvus* (yellow/orange cells).

To directly test the hypothesis that TraA is the specificity determinant, we replaced the *traA* allele in a *M. xanthus* laboratory strain to investigate possible cognate changes in strain recognition. As reported above (Fig. 1B), the wild-type *M. fulvus* and *M. xanthus* DK1622 laboratory strains do not transfer OM components (Fig. 4; top panels). Importantly, when an isogenic *M. xanthus* strain expressed the *traA^M. fulvus^* allele, it was able to partner with *M. fulvus* for efficient transfer (Fig. 4; bottom panels). Similarly, when the *traA* alleles from strains DK816, A96 and Pali were used to replace the *traA* allele in the laboratory strain, we observed a corresponding change in partner transfer specificity (data not shown). In addition, when merodiploid strains were constructed that contained two alleles of *traA*, transfer occurred between both recognition groups, showing that multiple *traA* alleles broaden host range recognition and that the alleles are not antagonistic (data not shown).

### TraA exhibits allele-specific interactions

**Figure 5:**
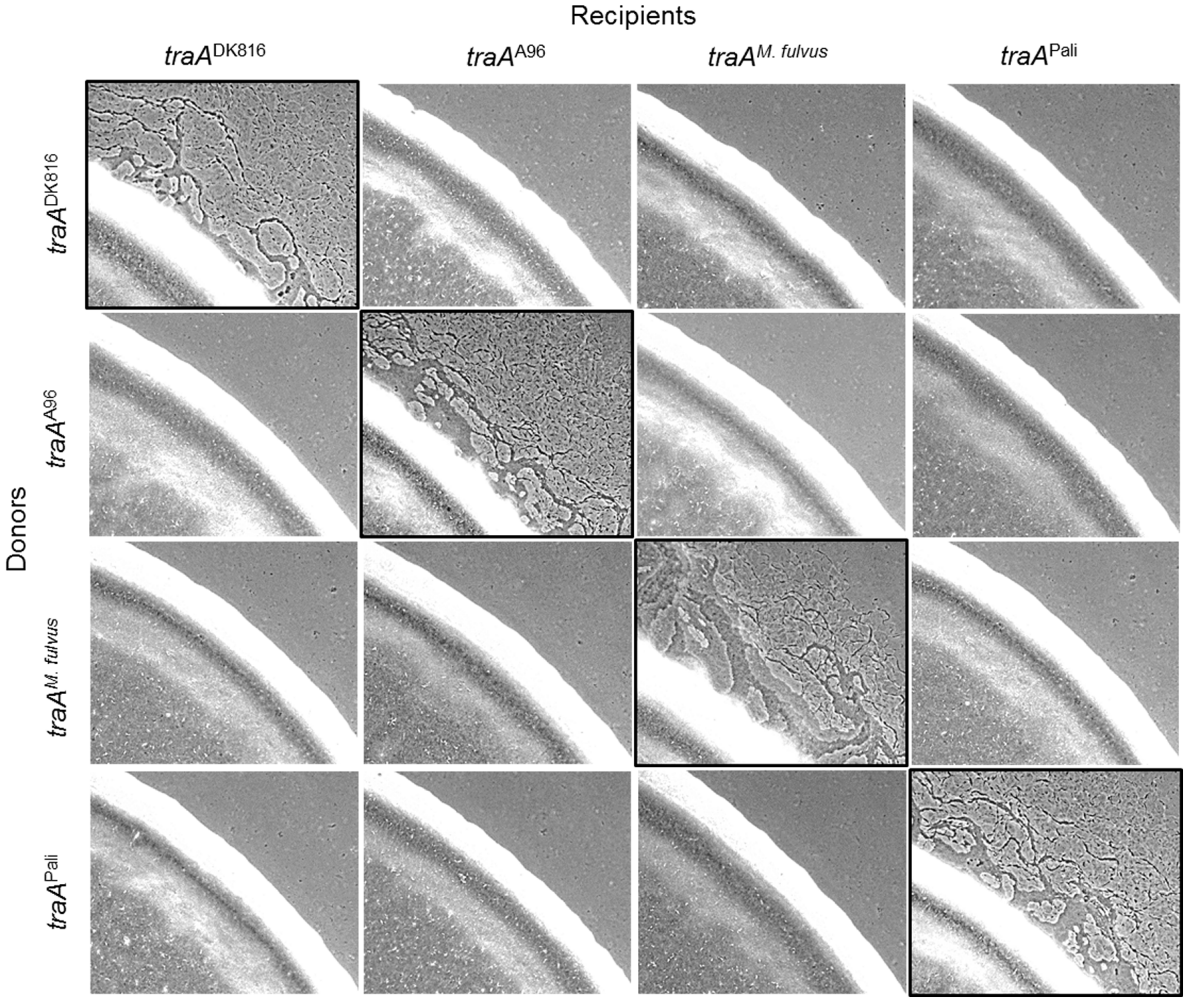
*traA* allele–specific interactions in extracellular complementation of gliding motility. Protein transfer was assayed by the ability of nonmotile recipient mutants (Δ*cglC* Δ*tgl*) to be complemented extracellularly by a nonmotile, nonstimulatable donor that encodes the wild-type CglC and Tgl proteins [7]–[9]. The four engineered donor strains encoded the indicated *traA* allele replacements. The recipient strains were merodiploid with the indicated *traA* alleles and the original *traA* ^DK1622^ allele. Strains were mixed at a 1∶1 cell ratio, and micrographs were taken after 1 day. Images with black borders show *traA* allele combinations that restore motility, which occurs by protein transfer [9]. Strains are listed in Table S1.

To substantiate the above findings, we used an extracellular complementation (stimulation) assay that phenotypically assesses protein transfer. In this assay certain nonmotile mutants (recipients) can have their motility defect rescued by the transfer of functional proteins from donor cells that encode the corresponding wild-type protein [7], [10], [15]. In these experiments, four isogenic nonmotile and nonstimulatable donor strains were constructed with the indicated *traA* allele replacements (Fig. 5). Similarly, four isogenic nonmotile stimulatable recipients (laboratory strains; Δ*cglC* Δ*tgl*) were constructed with the identical set of *traA* alleles. These eight strains were mixed in all possible combinations between donors and recipients. After 1 day of incubation, phenotypic rescue, as judged by emergent colony flares, had occurred only when donors and recipients expressed the same *traA* allele; phenotypic rescue did not occur between alleles from different recognition groups (Fig. 5). Therefore, in isogenic strain backgrounds, TraA interactions are allele specific, and these results are in perfect agreement with the above recognition group designations (Fig. 1B).

### TraA governs population behaviors by an allele-specific mechanism

Myxobacteria are unusual because they exhibit complex and coordinated behaviors that are typically not found in other bacteria. Recently, we discovered that Tra-dependent OM exchange regulates a new form of cell-cell interactions. These interactions were uncovered when genetically distinct strains were mixed and it was found that one strain could regulate the behavior of another strain in terms of motility and development behaviors. In particular, nonmotile strains can prevent swarm expansion and fruiting body formation of motile strains [5], [7], suggesting that TraAB-catalyzed OM fusion forms a communication conduit between cells. Because myxobacteria must coordinate their behaviors, any reduction in this ability to coordinate behaviors should result in reduced fitness in those individuals. The nature of the proposed signal(s) produced in the nonmotile strain that blocks swarming in the motile strain is unknown, although it clearly is not a diffusible signal [5].

**Figure 6:**
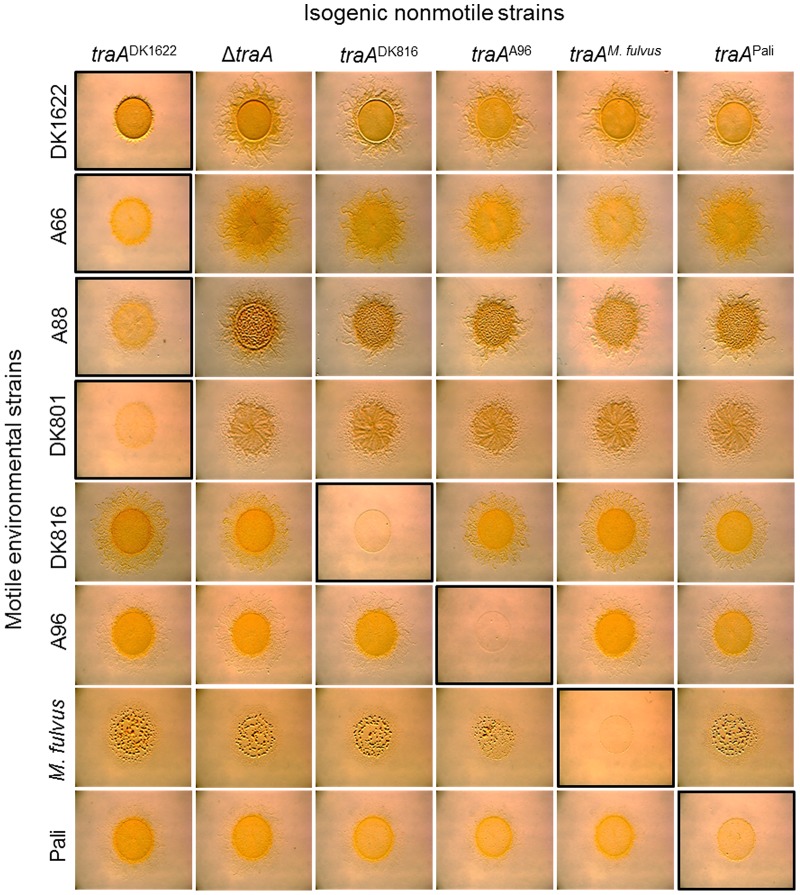
*traA* allele–specific regulation of swarming. Indicated motile strains were mixed with isogenic engineered nonmotile laboratory strains that encoded the indicated *traA* alleles. Mixtures in which both strains encoded identical *traA* alleles or belonged to the same recognition group are highlighted with a black border, and they all exhibited swarm inhibition. A nonmotile Δ*traA* strain (DW1467) was used as a negative control (full swarming). Stereomicrographs were taken after 2 days of incubation. Assay was done as described [7].

Here we sought to extend those findings to test whether the TraA recognition groups defined above could predict the outcomes for inter-strain swarm regulation. To do this we again tested for *traA* allele–specific interactions between nonmotile and motile strains. In these experiments six isogenic strains were constructed wherein each strain contained different environmental *traA* alleles. These strains were mixed at a 1∶1 ratio with eight different environmental isolates that were fully motile (adventurous and social motility; A^+^S^+^) and placed on swarm agar surfaces and allowed to swarm for 1 day. As was previously reported [7], a nonmotile *traA* ^DK1622^ strain blocked swarm expansion of the motile DK1622 strain (Fig. 6, top left panel). Importantly, DK1622 swarm inhibition was allele specific, as a Δ*traA* strain or any of the four other *traA* allele replacement strains that were not from recognition group A resulted in no swarm inhibition (Fig. 6, top row). In agreement with Figure 1B findings, the nonmotile *traA* ^DK1622^ strain also specifically blocked swarm expansion of all other group A members (A66, A88 and DK801) but not of members from other recognition groups (Fig. 6, left column). Moreover, the engineered *traA* ^DK816^, *traA* ^A96^, *traA^M. fulvus^* and *traA* ^Pali^ nonmotile strains specifically blocked swarm expansion of their cognate motile strains, i.e., DK816, A96, *M. fulvus* and Pali, respectively, but not of strains that belonged to different recognition groups (Fig. 6). These results therefore indicate that *traA* regulates the decision of the population to swarm in an allele-specific manner.

### TraA confers immunity from inter-strain killing

**Figure 7:**
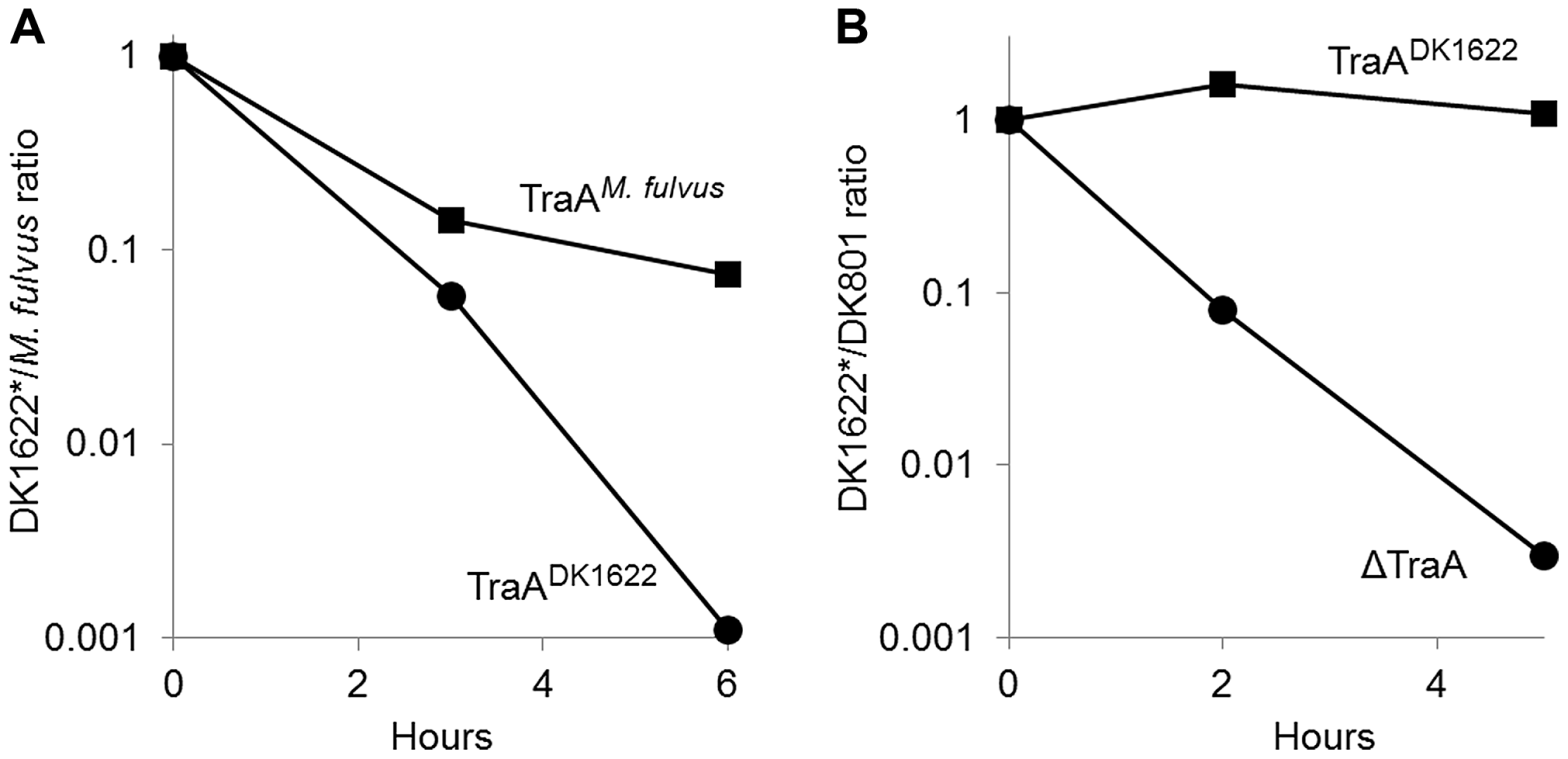
TraA-dependent OM exchange confers protection from inter-strain killing. A) Labeled *M. fulvus* (stained red with DiD lipid dye) was mixed at a 1∶1 cell ratio with isogenic labeled DK1622 derivative strains (stained green with CFDA SE) that contain either TraA^DK1622^ (DK8601*) or TraA*^M. fulvus^* (DW1470*). After incubation on an agar surface for the indicated times, cells were collected for microscopic examination to determine the ratio of red to green or yellow cells. Between 300 and >1,000 cells were scored for each time point. B) The experiment was carried out as in A, except DK801 was mixed with isogenic DK1622 derivative strains that contained either TraA^DK1622^ (DK8601*) or ΔTraA (DW1467*). Results are representative from multiple experiments.

Myxobacteria are highly antagonist toward nonself microbes, and they prey on other bacteria and fungi for food [16]. Myxobacteria also produce growth inhibitory or lytic substances (bacteriocins) that act specifically against other myxobacterial strains [17]–[19]. Consistent with these earlier observations, during the course of strain mixing experiments (Fig. 1B), we found evidence that certain isolates killed other strains. For example, when we mixed green and red fluorescently labeled strains, we sometimes observed that one or both of the isolates would contain some cells that lysed during the ∼4-hr incubation. Because TraA facilitates the transfer of many OM components, which may include hundreds of different proteins [7], [8], we tested whether OM exchange might regulate antagonistic interactions between *Myxococcus* strains. Similar to some of the other environmental isolates, *M. fulvus* was found to kill the laboratory strain (DK1622 or derivatives). This was first observed when red-labeled *M. fulvus* cells were mixed with a green-labeled *M. xanthus* strain at a 1∶1 ratio (Fig. 7A). After 6 hr of incubation, green-labeled DK1622 derivative cells were not detected. In contrast, thousands of red *M. fulvus* cells were easily detected. We further quantified *M. xanthus* killing by plating cells from such mixtures to determine viable colony forming units (CFU) and found that after 1 day of incubation no viable DK1622 derivative cells were found (>7 log killing; Fig. S3). To test whether killing was influenced by OM exchange, an isogenic *traA^M. fulvus^* allele replacement strain that transferred with *M. fulvus* (Fig. 4) was instead used. Interestingly, heterologous expression of TraA*^M. fulvus^* was indeed found to confer protection to *M. xanthus* (DK1622 derivative) from *M. fulvus* killing, although the protection was not absolute (Fig. 7A). Similarly, when we tested for CFU viability after 1 day of co-incubation, the *M. xanthus* TraA*^M. fulvus^* strain showed a significant increase in survival, i.e. from no detectable survivors for the Tra*A* ^DK1622^ strain to >10^4^ CFU for the TraA*^M. fulvus^* isogenic strain (Fig. S3). These results show that heterologous expression of a cognate recognition group *traA* allele confers protection from killing.

In a reciprocal experiment, we sought to test if inactivating OM exchange between natural recognition group members would have an effect on inter-strain killing. For this analysis, we compared killing between *M. xanthus* recognition group A members DK801 versus DK1622, in which the latter strain either expressed a cognate *traA* ^DK1622^ allele or contained a Δ*traA* allele. Unlike what was found for the *M. fulvus*/DK1622 strain mixture, DK801/DK1622 mixtures co-existed in a relatively harmonious relationship, as the ratio between the strains remained near one during the time course of the experiment (Fig. 7B). In contrast, when the ΔTraA strain was instead mixed with DK801, its viability sharply decreased, with approximately 1,000-fold fewer cells, as compared with the isogenic TraA^DK1622^ strain (Fig. 7B). Thus inactivation of OM exchange within a natural recognition group can affect inter-strain killing. We hypothesize that OM exchange facilitates the transfer of an immunity factor(s) to the susceptible strain, which in turn protects that strain from killing by a bacteriocin or toxin.

## Discussion

**Figure 8:**
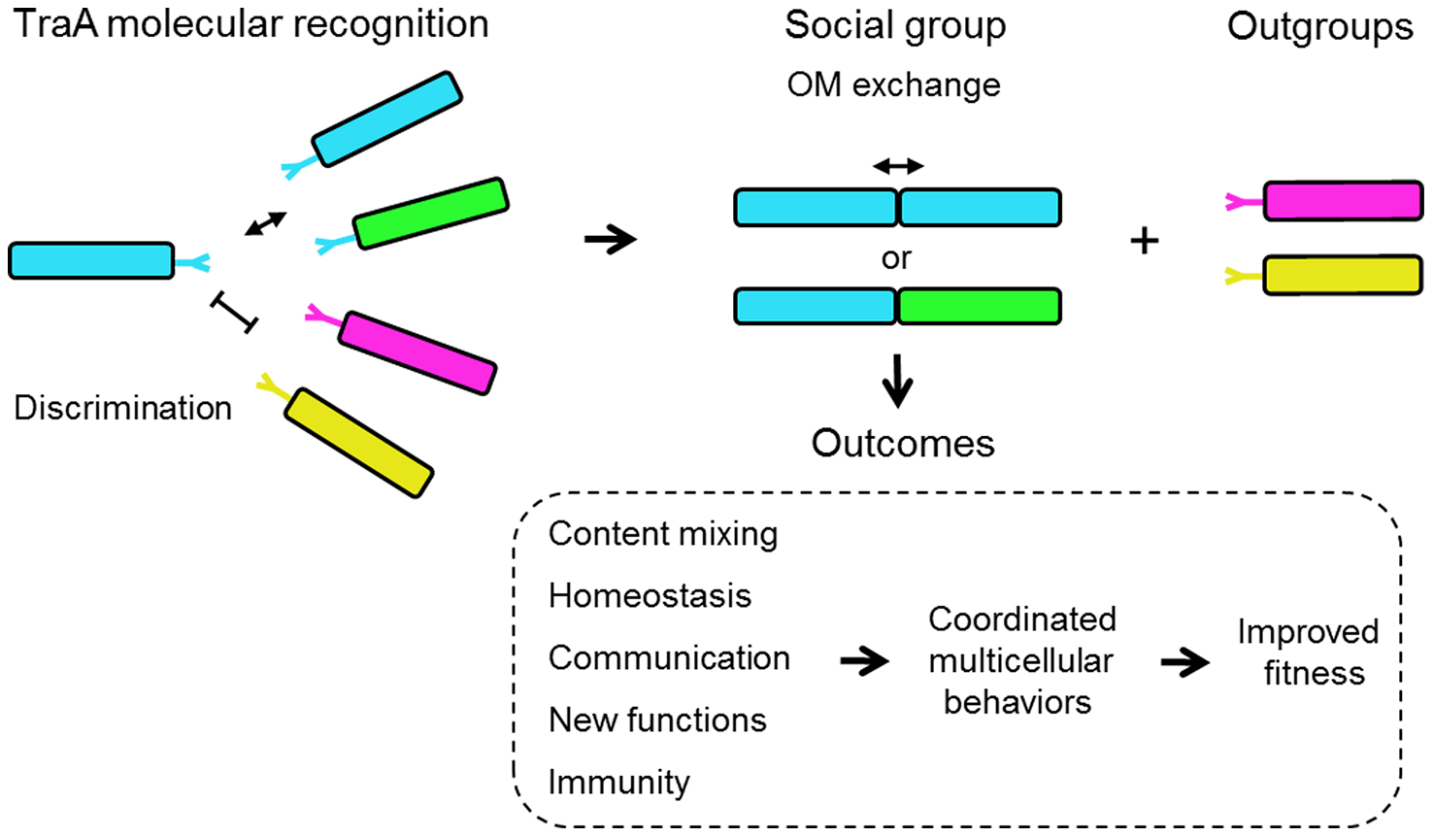
Schematic overview for how TraA-mediated cell-cell interactions can contribute toward myxobacterial social behaviors. Cell genotypes and TraA receptors are color coded to indicate genetic relatedness. Related TraA receptors bind through proposed homophilic interactions. Populations of low genetic diversity would likely result in only sibling interactions, whereas diverse populations could result in non-kin interactions and could contribute toward group selection dynamics [45]. Subsequent OM fusion and component exchange results in the indicated social outcomes. The ability of non-kin cells to interact could result in positive fitness outcomes. For example, if two distinct *M. xanthus* populations are of insufficient size to build a fruiting body, their combined populations, as mediated by TraA interactions, may be able to surmount this barrier.

Our results indicate that TraA functions as a polymorphic cell surface receptor that mediates cell-cell recognition for OM exchange. The simplest interpretation for how specificity occurs is that TraA binds identical or similar copies of itself on neighboring cells through homophilic interactions (Fig. 8). In particular it is the distant PA14-like domain within TraA that encodes the predictive features for recognition (Fig. 2B). Therefore our current model differs from our prior model that postulated the distant PA14 lectin-like domain of TraA binds glycans on neighboring cells [7], [20]. In addition, since *traA* allele replacements were necessary and sufficient to reprogram partner recognition (Fig. 5), it suggests that TraB is not a specificity factor. Moreover, since the *traB* ^DK1622^ allele functioned with four divergent *traA* alleles (Fig. 5), it seems that if TraA and TraB physically interact, as we have suggested [7], they do so between conserved residues within TraA (Fig. 2A). Importantly, the ability of TraA to discriminate between partnering cells supports our original hypothesis that OM exchange, which results in sharing of substantial cellular resources, is a regulated and selective process.

Bacterial molecular recognition is an emerging field of study and a number of interesting examples have been described in various levels of detail [21]–[24]. Typically these systems function in adhesion and/or toxin/immunity interactions. However, to our knowledge there is no other example of a bacterial recognition system that is involved in complex cooperative behaviors such as those described here for TraA. In turn OM exchange has broad implications for social interactions among myxobacteria (Fig. 8). For instance, we propose it provides a communication conduit to coordinate social functions, such as the decision of a population to swarm (Fig. 6). Importantly, the exchange of hundreds of different proteins [8] also allows cells to transiently repair/replace OM proteins that have been damaged by environmental and genetic insults (Fig. 5), obtain new protein functions (Figs. 7 and S3) and to strive toward population OM homeostasis by equilibrating component levels. This interpretation has striking similarities to the explanation given for mitochondria dynamics. Here, investigators have proposed that mitochondria undergo rounds of fusion and fission to mix and repair organelle components to establish a coherent and functional population [25], [26].

One important difference between myxobacteria and mitochondria fusion/fission is that the former represents independent cells, while the latter occurs within the confined space of a single cell. In this context myxobacteria can express different proteins because the cells are not necessarily siblings nor originated from the same micro-environments. Thus myxobacteria OM exchange may provide new functions. In a dramatic display of new function, we found that strain protection from inter-strain killing can be transferred (Fig. 7). Presumably inter-strain protection occurs by the transfer of immunity factors between cells. Consistent with this idea, bioinformatic analysis has found that *Myxococcus* genomes encode many toxin/immunity factor pairs [27]. Future studies will need to elucidate the details of how inter-strain killing and TraA-mediated protection works, and such a determination might be challenging, as myxobacteria produce cocktails of anti-microbial agents [16], [17], [27].

Unfortunately, our understanding of how myxobacteria—or for that matter most bacteria—actually live and interact in their environments is poor. However, one ecological study did investigate to what extent *M. xanthus* strains vary within a soil sample [28]. Based on molecular and phenotypic analyses at a centimeter scale resolution, this myxobacterial community was heterogeneous, as the 78 isolates parsed into at least 45 distinctive strains [29]. Five of these strains were used in our study (A23, A47, A66, A88 and A96). These strains represent two distinct genotypes, as defined by multilocus sequence typing (MLST), and our *traA* sequence analysis indeed showed that A66/A88 and A47/A96 have identical *traA* sequences, respectively, as would be expected from the MLST results. In contrast, although A23 belongs to the previously defined A47/A96 genotype, its *traA* sequence was significantly divergent from that of A47/A96, and it functionally belongs in a distinctive recognition group (Figs. 1B and 2B). Given that local *M. xanthus* communities are genetically diverse, the ability of clonal groups to recognize self from nonself would presumably be critical for their social interactions and for the transition into a multicellular fruiting body. Based on our studies, we suggest that TraA represents one molecular mechanism for kin recognition (Fig. 8). We also predict that myxobacteria have other recognition mechanisms [18], [29]–[31].

Our results suggest that TraA functions as a molecular determinant for self/nonself recognition. As sibling cells necessarily express identical *traA* alleles, they would form a kin recognition group. However, as Figure 1B illustrates non-kin cells can also belong to the same group (Fig. 8). Although not obviously revealed in Figure 1B, in mechanistic terms the relative affinities of TraA receptors within a given recognition group may vary between alleles, such that kin interactions might be preferred. For example, it is possible that receptors with identical sequences may form higher-affinity interactions than those between recognition members with more divergent TraA sequences. In one case, we did observe this: a low level of exchange was observed between *M. fulvus* and Mxx23 (Fig. 1B). In general, however, our assays likely provide a low-resolution assessment of relative binding affinities, and thus moderate and high-affinity interactions may yield similar outcomes. In contrast, cells in the environment might interact under less favorably conditions than laboratory conditions, where binding affinities may play a stronger discriminatory role. To test the hypothesis that TraA affinities vary within recognition groups, a more quantitative assay will need to be developed.

An alternative idea is that promiscuous interactions within recognition groups are functionally important. For example, promiscuous interactions could assist myxobacterial communities to reach the critical number of cooperative cells needed for fruiting body development. This numerical requirement that hundreds of thousands of cells must unite to build a viable fruit is a daunting threshold given the sparse growing conditions associated with microbial life in the soil. Thus the ability of non-kin cells to combine their resources and cell numbers to build a fruit may ease this transition. From our experience, we think the major obstacle in combining inter-strain resources is inter-strain killing (Fig. 7) [18]. As shown here, the formation of functional recognition groups partly alleviates the propensity of myxobacteria strains to kill one another (Fig. 7).

A fundamental question in evolutionary biology is how cooperative social behaviors evolved in the context of seemingly contradictory Darwinian evolution [32]. The ‘greenbeard’ concept, in which a single gene allows individuals to provide preferential treatment toward others, provides a tangible framework for how cooperation could evolve [1], [33], [34]. This abstract concept was refined by Haig, who explained it in molecular terms [35]. He proposed that a homophilic cell surface receptor could fulfill the three greenbeard requirements: it is a feature or trait, it allows recognition in others of the same gene product, and it results in cooperative behavior or ‘nepotism’ toward those individuals. The greenbeard concept differs from kin selection in that the helping behavior is directed toward other individuals with the same greenbeard gene, regardless of the genetic relatedness between individuals. Our described properties of *traA* represent a rare case in which a single gene meets these greenbeard criteria. That is, our evidence suggests that TraA functions as homophilic receptor/adhesin that recognize other cells that bear a genetically related allele, irrespective of kin relationships, to catalyze OM fusion that results in beneficial social outcomes (Fig. 8). This TraA-dependent form of nepotism allows cell-cell signaling and cellular resource sharing to occur selectively (Fig. 8). As mentioned, a dramatic display of TraA-dependent greenbeard nepotism is protection from killing. As myxobacteria apparently have many forms of toxin/immunity systems in their genomes [27], TraA can potentially provide an umbrella protection platform that circumvents toxin(s) action specifically to cognate recognition group members. Another implicit requirement of greenbeard genes, which we have shown for TraA, is that their sequences must be polymorphic, which provides a mechanism for selective recognition among individuals. To our knowledge, TraA is the first helping greenbeard (single) gene described in bacteria. In yeast and the soil amoeba *Dictyostelium discoideum*, which similarly transitions from free-living cells into multicellular fruiting bodies, there are other examples of greenbeard genes that police social interactions [36]–[38].

Because OM exchange affects a wide variety of cellular functions, the question arises as to whether there is a single driving benefit that TraA is being selected for in the environment. In a foreshadowing discussion to this work, Haig suggested that myxobacteria use greenbeard recognition in ‘security surveillance’ to identify friend from foe for multicellular development [39]. Haig argued that such a system would prevent exploitation of somatic cells (terminally differentiated cells that autolyse or form stalk cells) by germ line cells (spores) during fruiting body formation [40]. Whether TraA plays a role in surveillance recognition during development remains to be investigated.

We suggest that the TraA greenbeard concept provides clues for the functional and evolutionary transitions from single cell to multicellular life (Fig. 8). Specifically, TraA confers cell recognition that leads to cell-cell communication and sharing of otherwise private cellular goods. In turn, a cell population can transition from a phenotypically heterogeneous collection of individual cells into a tissue-like state of homeostasis, which refines and promotes cooperative multicellular interactions.

## Materials and Methods

### Bacterial strains and growth conditions

Table S1 lists bacterial strains and plasmids used in this study. Routine cloning was done in DH5α *Myxococcus* cultures were grown to a Klett reading of ∼100 (3×10^8^ colony forming units [CFU]/ml) at 33°C in CTT medium (1% casitone, 1 mM KH_2_PO_4_, 8 mM MgSO_4_, 10 mM Tris-HCl, pH 7.6) in the dark; when necessary, cultures were supplemented with kanamycin (Km; 50 µg/ml) or oxytetracycline (Tc; 15 µg/ml). For ½ CTT, casitone was reduced to 0.5%. On plates, the agar concentration was 1.0 or 1.5%. TPM buffer contains 10 mM Tris, 1 mM KH_2_PO_4_ and 8 mM MgSO_4_ (pH 7.6). *Escherichia coli* cultures were grown at 37°C in LB medium and, when necessary, were supplemented with Km (50 µg/ml) or ampicillin (Ap; 100 µg/ml).

### Cell staining and transfer assay

GFP and mCherry reporters were used to monitor transfer as described [8]. In addition, as genetic transformation of environmental strains proved difficult, a new method was also developed. Here a red fluorescent DiD lipid dye (Lipophilic Tracer Sampler Kit; Invitrogen) vial H (5,5′-Ph2-DilC_18_(3)) was used to label *Myxococcus* OMs. These cells are referred to as donors, as this dye can be transferred via a Tra^+^-dependent mechanism [7]. Vial H worked best, as it effectively stained cell membranes, was bright under a Texas Red-4040B (Semrock) filter set and did not fluoresce under the FITC filter set (data not shown). Log phase cultures were collected by centrifugation and resuspended in TPM to a calculated density of 8×10^8^ CFU/ml. Then 2 µl of dye (1 mg/ml in ethanol) was added to 98 µl of the cell suspension and incubated for 1 hr at 33°C with occasional gentle vortexing. Cells were washed twice with 1 ml TPM and were ready to be mixed with recipients. Recipient strains were labeled in their cytoplasm with Vybrant CFDA SE (carboxyfluorescein diacetate, succinimidyl ester) Cell Tracer Kit (Invitrogen). Briefly, concentrated log phase cells (∼1.5×10^9^ CFU/ml) were stained for 30 min with CFDA SE dye following the manufacturer's instructions, except TPM was used instead of PBS. Cells were then washed twice with TPM and resuspended in 400 µl ½ CTT and incubated at 33°C for 1 hr with occasional gentle vortexing. During this incubation, cells enzymatically convert the nonfluorescent molecule into a green fluorescent derivative that is trapped in the cytoplasm as reactive products form fluorescent conjugates with intracellular amines (e.g., proteins) (Invitrogen). Live stained recipients were then washed three times in TPM and mixed at a 1∶1 cell ratio with live red donors and pipetted onto ½ CTT 1.5% agar plates. After 2–4 hr of incubation at 33°C, cells were collected from the plates and washed twice in TPM. The cells were then mounted on polysine-coated slides for microscopic examination [7], [8]. Transfer was scored by the ability of green recipient cells to obtain red fluorescence. Three micrographs, phase contrast and red and green fluorescence, were taken for each viewing field, and the red and green fluorescence images were subsequently merged with Image-Pro Plus software (Media Cybernetics). Transfer was scored as positive when the majority (usually >80%) of the recipients were red (yellow/orange in merged images). Transfer was scored as negative when ≤1% of the recipients were red. In a few cases, 10–20% of the recipients were positive and thus were scored as ‘±’ for poor transfer. Similar to prior reports, transfer specifically occurred between motile strains and required a biofilm on a hard surface and TraA function [7], [8]. We note two difficulties with this relatively time-intensive assay. First, mixing wild-type *Myxococcus* isolates often resulted in severe cell clumping. Second, inter-strain killing also occurred. For these reasons, experiments were repeated two or three times, and the corresponding reciprocal strain transfer was also typically tested to confirm the results.

### DNA sequencing of *traA* alleles

Genomic DNA was purified from cultures with a PureLink Genomic Kit (Invitrogen). The indicated *traA* alleles were PCR amplified with Taq Master Mix (New England BioLabs), and PCR reactions were gel purified with a QIAquick Gel Extraction Kit (Qiagen) and directly sequenced (Nucleic Acid Exploration Facility at University of Wyoming). Primers are listed in Table S2. The *traA* allele sequences were deposited in GenBank with the accession numbers JX876748–62.

### Plasmid and strain construction

To construct the *traA* deletion cassette, regions upstream and downstream of the gene were PCR amplified from the DK1622 chromosome, digested with appropriate restriction enzymes and cloned into pBJ114 to generate pDP28. This plasmid contains a positive-negative Km^r^-*galK* selection cassette. pDP28 was electroporated into the strain DK8601 (*aglB1*, *pilA*::Tc), and a Km^r^ transformant was subsequently counter-selected on 1% galactose CTT agar. The Δ*traA* allele was confirmed by PCR with primers flanking the deletion site to generate DW1467. To create a *traA* allele replacement plasmid, the strong *pilA* promoter was first amplified with Phusion High-Fidelity DNA Polymerase (New England BioLabs) and cloned into pSWU19 at the *Eco*RI and *Xba*I restriction sites to make pDP22. Next, various *traA* alleles with an engineered ribosomal binding site were similarly PCR amplified from indicated isolates and cloned (*Xba*I and *Hind*III) downstream of the *pilA* promoter in pDP22 to generate plasmids pDP23–27 (Table S1). Verified plasmids were transformed into DW1467 and homologously integrated into the genome by selecting for Km^r^. To generate TraA antigen, the domain encoding PA14^DK1622^ was PCR amplified with Phusion and cloned into pMAL-c2X (New England BioLabs) at the *Eco*RI and *Pst*I sites (pDP29). All primers are listed in Table S2.

### Immunological methods

A protease-deficient *E. coli* strain (*clpX* ^−^ *clpY* ^−^ *lon^−^*) harboring pDP29 was grown in LB and induced at an OD_595_ of 0.6 with 1 mM IPTG for 4 hr at 37°C. Cells were harvested by centrifugation, lysed with a French press and pulse sonicated. Cell debris was removed by centrifugation (20,000×*g*), and the resulting supernatant was passed through a 0.2-µm PES filter (Whatman) to obtain a clear suspension. Soluble material was loaded onto a 5-ml HiTrap column connected to an ÄKTAprime chromatography system (GE Healthcare Life Sciences) for purification of maltose binding protein (MBP) fusion. Purification was carried out following the manufacturer's instructions. Protein concentration was determined by a Bradford assay (Thermo Scientific). About 10 mg of purified MBP-PA14 protein was sent to a commercial vendor (Thermo Scientific Pierce Protein Research) as antigen. Prior to immunization, pre-immune sera from five rabbits were pre-screened by western blot analysis against *M. xanthus* whole-cell extracts to select two rabbits that exhibited minimal background cross-reactivity. Proteins were separated by 10% SDS-PAGE and transferred to a polyvinylidene difluoride membrane as described [41]. Primary PA14 antibody was used at a 1∶30,000 dilution, and a secondary horseradish peroxidase–conjugated goat anti-rabbit antibody was used at a 1∶15,000 dilution (Pierce).

For immunofluorescence studies, cells were grown to mid-log phase, harvested by centrifugation and washed in TPM. Cells (5×10^8^) were then resuspended in 1 ml TPM containing 2% BSA. Following 30 min of incubation with gentle shaking at room temperature (RT), primary antibody (1∶5,000 dilution) was added and further incubated for 45 min. Cells were then pelleted by centrifugation and washed four times with 1 ml TPM. After the cell pellet was resuspended in 150 µl TPM with 2% BSA, 1 µl of secondary antibody (1∶150 dilution; DyLight 488–conjugated donkey anti–rabbit IgG; Jackson ImmunoResearch) was added and incubated for 45 minutes at RT. After incubation, cells were pelleted and washed four times in TPM. The labeled cells were mounted and examined with a fluorescence microscope equipped with a 100× phase contrast oil objective lens. To determine cell viability after processing for microscopic examination, total cell numbers were counted in a hemocytometer chamber (Hausser Scientific), followed by 10-fold serial dilutions and plating on CTT agar. After 5 days of incubation at 33°C, CFUs were determined. After processing for immunofluorescence imaging, cells were found to be 100% viable.

### Phylogenetic analysis

Sequences were aligned with MUSCLE [42], and model testing was performed using ProtTest [43]. Based on the best ProtTest model with four gamma categories, the tree was constructed by MrBayes3 software [44]. The tree was run for 10,000,000 generations, and the consensus tree was constructed with a default (25%) burnin phase.

### Microbial assays

Stimulation and swarm inhibition assays were essentially done as described [7]. For the swarm inhibition assay, no CaCl_2_ was added to ½ CTT agar. For interspecies kill assays, cultures were grown to mid-log phase, harvested by centrifugation and resuspended to a calculated density of 3×10^9^ CFU/ml. Cultures were mixed together (50 µl of each), and four 25-µl spots were placed on ½ CTT/2 mM CaCl_2_/1.5% agar plates. After 24 hr of incubation at 33°C, spots were harvested in 1 ml of TPM, vortexed for 20 sec and repeat pipetted (∼10 times). To further break up small clumps, cells were transferred to a sterile 1-ml glass tissue homogenizer and slowly plunged 10 times. Samples were then serially diluted in TPM, and 10-µl spots were placed on CTT and CTT Km agar plates. Plates were inspected daily for about a week to enumerate CFUs. *M. fulvus* colonies were identified on CTT plates as swarm proficient; the *M. xanthus* strains were nonmotile and Km^r^ and were enumerated on CTT Km plates. All experiments were carried out in triplicate, and the resulting values were averaged. In a second approach, interspecies killing was assayed by labeling respective strains with red or green fluorescence markers as described above. Such strains were mixed and pipetted onto ½ CTT agar. At various time points, cells were harvested, and the relative ratios of red and green labeled cells were microscopically determined.

## Supporting Information

Figure S1 Sequence alignment of the PA14 hyper-variable regions from 17 environmental isolates of myxobacteria, as generated with MUSCLE default settings [42]. Alignments start at the first residue after the predicted signal sequence (SS) cleavage site (Fig. 2B). For reference, the TraA^DK1622^ sequence spans positions 37 to 305. Invariant residues are indicated with asterisks.
(DOCX)

Figure S2 Evidence that horizontal DNA transfer and rearrangement occurred among ancestral *traA* alleles. TraA protein alignments were generated by MUSCLE default settings. Residues shared by two sequences are color coded. The site where a proposed recombination event occurred in the corresponding gene is marked by a black arrow. See Figure 2C for transfer specificity.
(DOCX)

Figure S3 TraA-dependent OM exchange confers immunity from inter-species killing. *M. fulvus* was mixed at a 1∶1 cell ratio with isogenic nonmotile *M. xanthus* strains that contained either TraA^DK1622^ (DW1476) or TraA*^M. fulvus^* (DW1470); the cells were incubated for 24 hr on agar prior to determining the cell viability of each strain. Experiments were done in triplicate, averaged and standard errors plotted.
(PDF)

Table S1 Plasmids and strains used in this study.
(DOCX)

Table S2 Primers used in this study.
(DOCX)
