# Supplementary material for: Molecular Recognition by a Polymorphic Cell Surface Receptor Governs Cooperative Behaviors in Bacteria
Source: PLoS Genet. 2013 Nov 7;9(11):e1003891. doi: 10.1371/journal.pgen.1003891 (PMC3820747; doi:10.1371/journal.pgen.1003891)
Supplement: Figure S1 — Sequence alignment of the PA14 hyper-variable regions from 17 environmental isolates of myxobacteria, as generated with MUSCLE default settings [42]. Alignments start at the first residue after the predicted signal sequence (SS) cleavage site (Fig. 2B). For reference, the TraADK1622 sequence spans positions 37 to 305. Invariant residues are indicated with asterisks. (DOCX) [file pgen.1003891.s001.docx]

DK1622 QPEPGEKEPELVRIRGTPVAPSPGAVGTGLCMASSTSSNPAVDFSQSEATFPGTFNAFME

A66 QPEPGEKAPEIVRIRGTPVAPSPGAVGTGLCMASSTSSTPAVDFPQSEATFPGTFNAFME

A88 QPEPGEKAPEIVRIRGTPVAPSPGAVGTGLCMASSTSSTPAVDFPQSEATFPGTFNAFME

DK801 QPEPGEKAPELVRIRGVPVAPSPGAVGTGLCMASSTSSTPAVDFPQSEATFPGTFNAFME

Pali ---QDEKLPDVV-VQGPPIAPALAGDGQGLCVASNVWTRSRNEFPQARGTYLDGINGYLE

DK816 ---QDEKLPDVV-VQGPPFAPALAGDGQGLCVASNVWTRSRNEFPLARGTYLDGINGYLE

A23 -----QKLPDVV-IQGPPIAPALAGDGQGLCVASNVWTRSLNEFPQTRGTYLDAINGYLE

DK805 -----QKLPDVV-IQGPPIAPALAGDGQGLCVASNIWTRPANEFPQSRGTYIDGINGFIE

*M.fulvus* -----QKLPDVV-IQGPPIASPLAGDGQGLCVASNVWTRPLNEFPQSRGTYIDGINGFLE

DK836 -----QKLPDVV-IQGPPIAPALAGDGQGLCVASNVWTRPLNEFPQSRGTYIDGINGFLE

DK854 -----QKLPDVV-IQGPPIAPALAGDGQGLCVASNVWTRPLNEFPQSRGTYIDGINGFLE

Mxx23 -----QKLPDVV-IQGPPIAPALAGDGQGLCVASNVWTRPLNEFPQSRGTYIDGINGFLE

DK823 ---QDEKLPDVV-VQGPPIAPALAGDGQGLCVASNVWTRPLGAFPQSRGTYIDGINGFLE

DK852 ---QDEKLPDVV-VQGPPIAPALAGDGQGLCVASNVWTRPLGAFPQSRGTYIDGINGFLE

A47 -----QKLPDVV-IQGPPIAPALAGDGQGLCVASNVWTRPLNEFPQSRGTYIDGINGFLE

A96 -----QKLPDVV-IQGPPIAPALAGDGQGLCVASNVWTRPLNEFPQSRGTYIDGINGFLE

GVK897 -----QKLPDVV-IQGPPIAPALAGDGQGLCVASNVWTRPLNEFPQSRGTYIDGINGFLE

:* *::* :.* *.*.. .. * ***:**. : . *. : .*: . :*.::*

DK1622 --SSRPRRVTSVLRTLFDLSNNITLGDPNDPTIQQPSYGDFVNSVGS--CGKGGCA----

A66 --SSRPRRVTSVLRTLFDLSNNITLGDPNDPTIQQPSYGDFVNSVGS--CGRGGCA----

A88 --SSRPRRVTSVLRTLFDLSNNITLGDPNDPTIQQPSYGDFVNSVGS--CGRGGCA----

DK801 --SSRPRRVTSVLRTLFDLSNNITLGDPNDPTIQQPSYGDFVNSVGS--CGRGGCA----

Pali EPTSRQSRITTVMRTLFDLSNNLNDG-------RTLSYGDFTNQVTAPNCAAGGCSFSAI

DK816 EPANRQTRVTTVLRTQFDLSNNLNDG-------RTLSYGDFVGQETSAGCSTGGCSFNII

A23 EPTSRQTRVTTVLRTQFDLSNNLNDG-------RTLSYGDFVGQETSTGCSTGGCSFNII

DK805 EPASRQTRVTTVLRTQFDLSNNLNDG-------RTLSYGDFVSQVTAPECSIGGCTFNVI

*M.fulvus* EPASRQSRVTTVLRTPFDLSNNLNDG-------RTLSYGDFVNQVAAPGCSIGGCTFNVI

DK836 EPASRQSRVTTVLRTPFDLSNNLNDG-------RTLSYGDFVNQVTAPGCSIGGCTFNVI

DK854 EPASRQSRVTTVLRTPFDLSNNLNDG-------RTLSYGDFANQVTAPGCSIGGCTFNVI

Mxx23 EPASRQSRVTTVLRTPFDLSNNLNDG-------RTLSYGDFVNQVTAPGCSIGGCTFNVI

DK823 EPASRQSRVTTVLRTPFDLSNNLNDG-------RTLSYGDFANQVTAPGCSIGGCTFNVI

DK852 EPASRQSRVTTVLRTPFDLSNNLNDG-------RTLSYGDFANQVTAPGCSIGGCTFNVI

A47 EPASRQSRVTTVLRTPFDLSNNLNDG-------RTLSYGDFVNQVTAPGCSIGGCTFNVI

A96 EPASRQSRVTTVLRTPFDLSNNLNDG-------RTLSYGDFVNQVTAPGCSIGGCTFNVI

GVK897 EPASRQSRVTTVLRTPFDLSNNLNDG-------RTLSYGDFANQVTAPGCSIGGCTFNVI

:.* *:*:*:** ******:. * . *****... : *. ***:

DK1622 SPHPTFSSFGARFRGYINVQPQWVEVPLHFGLYADDAVSFVIYDLSQTPYQVINRPPQLG

A66 SPHPTFSSFGARFRGYINVQPQWVEVPLHFGFYADDAVSFVIYDLSQTPYQVINRPPQLG

A88 SPHPTFSSFGARFRGYINVQPQWVEVPLHFGFYADDAVSFVIYDLSQTPYQVINRPPQLG

DK801 SPHPTFSSFGARFRGYINVQPQWVEVPLHFGFYADDAVSFVIYDLSQTPYQVINRPPQLG

Pali SDNDASTPFVARFRGYLNVPPELTNQPIHFGYYTDDAISLVIYDLSQS-HAVINRPPELG

DK816 ADNDAFTPAVSRFRGYLNVTAELAAQPLHFGFYADDAISMVVYDRSQS-YTVINRPPQLG

A23 ADNDAFTPAVSRFRGYLNVTAELAAQPLHFGFYADDAISMVVYDRSQS-YTVINRPPQLG

DK805 EDNDAFTSFVSRFRGYLNITEDLVAQPVHFGFYADDGISLVIYDRSQG-YQVINRPPVLG

*M.fulvus* EDNDAFTPFASRFRGYLNVTADLAGQPLHFGFYADDGISLVIYDRSQG-YQVINRPPMLG

DK836 EDNDAFTPFASRFRGYLNVTADLAGQPLHFGLYADDGISLVIYDRSQG-YQVINRPPMLG

DK854 EDNDAFTPFASRFRGYLNVTADLAGQPLHFGLYADDGISLVIYDRSQG-YQVINRPPMLG

Mxx23 EDNDAFTPFASRFRGYLNVTADLAGQPLHFGYYVDDGISLAIYDRSQA-YQVINRPPVLG

DK823 EDNDAFTPFASRFRGYLNVTADLAGQPLHFGYYVDDGISLAIYDRSQA-YQVINRPPVLG

DK852 EDNDAFTPFASRFRGYLNVTADLAGQPLHFGYYVDDGISLAIYDRSQA-YQVINRPPVLG

A47 EDNDAFTPFTSRFRGYLNVTADLAGQPLHFGYYVDDGISLAIYDRSQA-YQVINRPPVLG

A96 EDNDAFTPFTSRFRGYLNVTADLAGQPLHFGYYVDDGISLAIYDRSQA-YQVINRPPVLG

GVK897 EDNDAFTPFASRFRGYLNVTADLAGQPLHFGYYVDDGISLAIYDRSQA-YQVINRPPVLG

: : :. :*****:*: : . *:*** *.**.:*:.:** ** : ****** **

DK1622 IASWRTTNTVVFERPGLYPVEILYAQVSEHSALEFSTFVGAFPDTERGASTDPIVKLNTA

A66 IASWRTTNTVVFERPGLYPVEILYAQVSEHSALEFSTFVGAFPDTERGASTDPIVKLNTA

A88 IASWRTTNTVVFERPGLYPVEILYAQVSEHSALEFSTFVGAFPDTERGASTDPIVKLNTA

DK801 IASWRTTNTVVFERPGLYPVEILYAQVSEHSALELSTFVGAFPDTERGASTDPIVKLNTA

Pali AATWRTSNSVTFMQPGLYAVEMLYSQINEHAALEMSMRTGSFMGFERPANQPPVVNLFSS

DK816 APTWRTTNSVTFSQPGIYAVEMLYSQVTEHAALEMSMRTGDFTNFERAANQPPVINLFSS

A23 APTWRTTNSVTFSQPGIYAVEMLYSQVTEHAALEMSMRTGAFTDFERAANQPPVVNLFSS

DK805 AATWRSTNSVTFPQAGLYAVEILYAQVTEHAALELSMETGPFTDFERAANQPPVINLFSS

*M.fulvus* FPTWRTTNSVTFNAPGLYAVEVLYTQVGEHAALEMSMLTGAFTDFERAANQPPVVNLFSS

DK836 FPTWRTTNSVTFNAPGLYAVEALYTQVGEHAALEMSMHTGAFTDFERAANQPPVINLFSS

DK854 FPTWRTTNSVTFNAPGLYAVEALYTQVGEHAALEMSMHTGAFTDFERAANQPPVINLFSS

Mxx23 FATWRTTNSVTFNAPGLYAVEMLYSQVGEHAALEMSMNTGAFTDFERAANQPPIINLFSS

DK823 FATWRTTNSVTFNAPGLYAVEMLYSQVGEHAALEMSMHTGAFTDFERAANQPPVINLFSS

DK852 FATWRTTNSVTFNAPGLYAVEMLYSQVGEHAALEMSMHTGAFTDFERAANQPPVINLFSS

A47 FATWRTTNSVTFNAPGLYAVEMLYSQVGEHAALEMSMHTGAFTDFERAANQPPVVNLFSS

A96 FATWRTTNSVTFNAPGLYAVEMLYSQVGEHAALEMSMHTGAFTDFERAANQPPVVNLFSS

GVK897 FATWRTTNSVTFNAPGLYAVEMLYSQVGEHAALEMSMHTGAFTDFERAANQPPVINLFSS

.:**::*:*.* .*:*.** **:*: **:***:* .* * . ** *. *:::* ::

DK1622 GFALAPPELFFHTEGGRPSFPDDINRCEQCNRQFANI

A66 GFVLAPPELFFHTEGGRPSFPDDINRCEQCNRQFANI

A88 GFVLAPPELFFHTEGGRPSFPDDINRCEQCNRQFANI

DK801 GFVLAPPELFFHTEGGRPSFPDDINRCEQCNRQFANI

Pali GFQLLRPAQFFQTENGILSFEGQPDRCEQCNRGNASL

DK816 GFQLLRPAQFFQTENGILSFAGQPDRCEQCNRGNANQ

A23 GFQLLRPAQFFQTENGILSFEGQPDRCEQCNRGNANQ

DK805 GFQLLRPAQFFQTENGILSFAGQPDRCEQCNRGNANS

*M.fulvus* GFELLRPAQFFQTENGLLSIPDQPDRCEQCNRGNANA

DK836 GFQLLRPAQFFQTENGILSFAGQPDRCEQCNRGNANS

DK854 DFQLLRPAQFFQTENGILSFEGQPDRCEQCNRGNANS

Mxx23 GFQLLRPAQFFQTENGILSFEGQPDRCEQCNRGNANL

DK823 GFQLLRPAQFFQTENGILSFAGQPDRCEQCNRGNANQ

DK852 GFQLLRPAQFFQTENGILSFAGQPDRCEQCNRGNANQ

A47 GFQLLRPAQFFQTENGILSFEGQPDRCEQCNRGNANQ

A96 GFQLLRPAQFFQTENGILSFEGQPDRCEQCNRGNANQ

GVK897 GFQLLRPAQFFQTENGILSFAGQPDRCEQCNRGNANQ

.* * * **:**.* *: .: :******* *.
