# Supplementary material for: Molecular Recognition by a Polymorphic Cell Surface Receptor Governs Cooperative Behaviors in Bacteria
Source: PLoS Genet. 2013 Nov 7;9(11):e1003891. doi: 10.1371/journal.pgen.1003891 (PMC3820747; doi:10.1371/journal.pgen.1003891)
Supplement: Figure S2 — Evidence that horizontal DNA transfer and rearrangement occurred among ancestral traA alleles. TraA protein alignments were generated by MUSCLE default settings. Residues shared by two sequences are color coded. The site where a proposed recombination event occurred in the corresponding gene is marked by a black arrow. See Figure 2C for transfer specificity. (DOCX) [file pgen.1003891.s002.docx]

DK1622 = DK836

DK1622 = *M. fulvus*

*M. fulvus* = DK836
