# Supplementary figures and images for: Molecular Recognition by a Polymorphic Cell Surface Receptor Governs Cooperative Behaviors in Bacteria
Source: PLoS Genet. 2013 Nov 7;9(11):e1003891. doi: 10.1371/journal.pgen.1003891 (PMC3820747; doi:10.1371/journal.pgen.1003891)

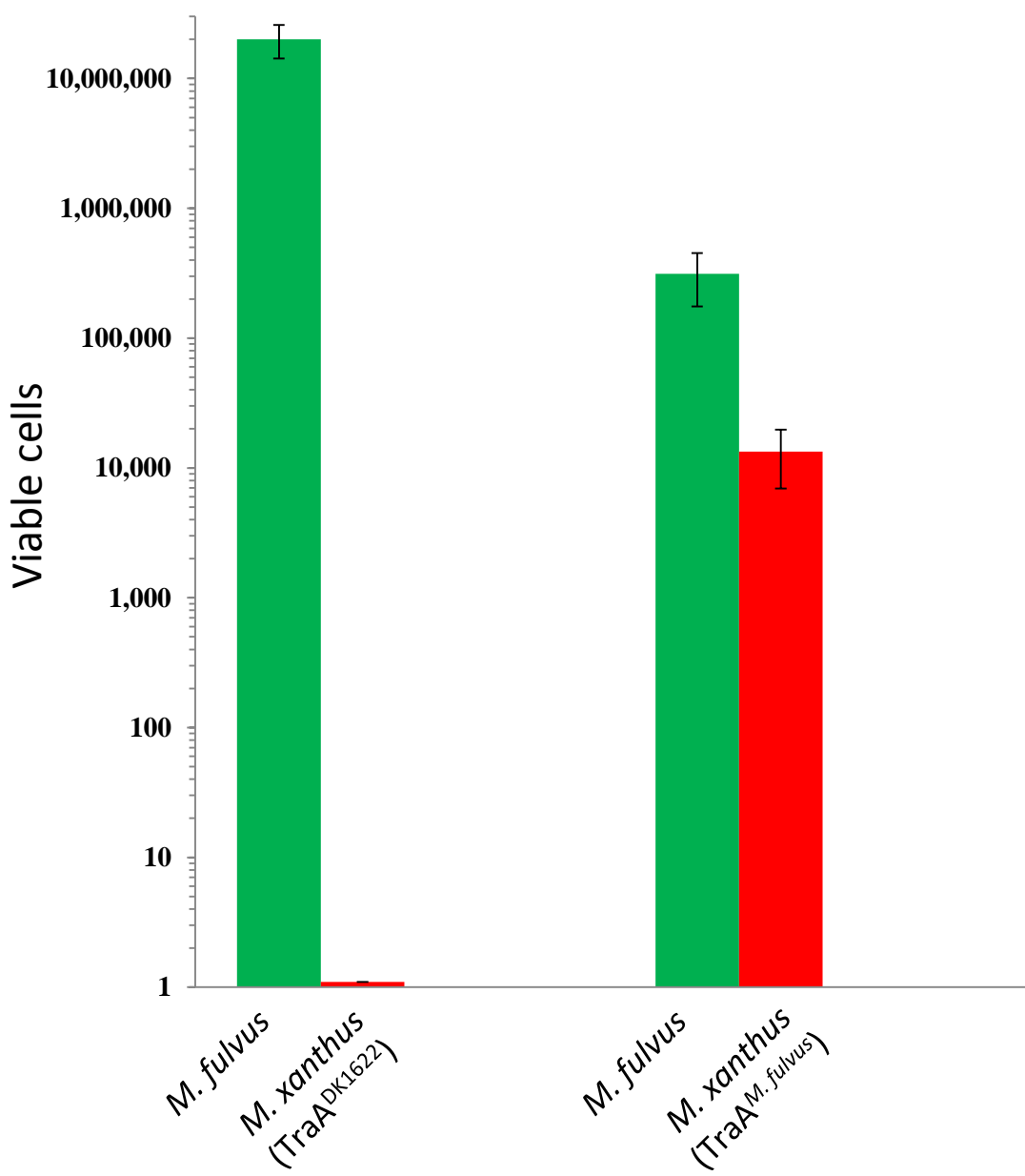

Supplement: Figure S3 — TraA-dependent OM exchange confers immunity from inter-species killing. M. fulvus was mixed at a 1∶1 cell ratio with isogenic nonmotile M. xanthus strains that contained either TraADK1622 (DW1476) or TraAM. fulvus (DW1470); the cells were incubated for 24 hr on agar prior to determining the cell viability of each strain. Experiments were done in triplicate, averaged and standard errors plotted. (PDF) [file pgen.1003891.s003.pdf]
