# Supplementary material for: Molecular Recognition by a Polymorphic Cell Surface Receptor Governs Cooperative Behaviors in Bacteria
Source: PLoS Genet. 2013 Nov 7;9(11):e1003891. doi: 10.1371/journal.pgen.1003891 (PMC3820747; doi:10.1371/journal.pgen.1003891)
Supplement: Table S1 — Plasmids and strains used in this study. (DOCX) [file pgen.1003891.s004.docx]

Table S1. Plasmids and strains used in this study.

| **Plasmid** | **Relevant features** | **Isolation/source/references** |
| --- | --- | --- |
| pDP22 | pSWU19 P*_pilA_*, Km^r^ | This study |
| pDP23 | pSWU19 P*_pilA_*-*traA*^DK816^, Km^r^ | This study |
| pDP24 | pSWU19 P*_pilA_*-*traA*^A96^, Km^r^ | This study |
| pDP25 | pSWU19 P*_pilA_*-*traA^M.fulvus^*, Km^r^ | This study |
| pDP26 | pSWU19 P*_pilA_*-*traA*^Pali^, Km^r^ | This study |
| pDP27 | pSWU19 P*_pilA_*-*traA*^DK1622^, Km^r^ | This study |
| pDP28 | pBJ114 Δ*traA* cassette, *galK* Km^r^ | This study |
| pDP29 | pMALc2X MBP-PA14^DK1622^, Ap^r^ | This study |
| **Strain** |  |  |
| DH5α | *E. coli* cloning strain | Lab collection |
| XF100 | *E. coli clpX*^–^ *clpY*^–^ *lon*^–^ | Gomelsky collection |
| A23 | Wild-type *M. xanthus* | Tübingen, Germany; [[28, 29](#_ENREF_2)] |
| A47 | Wild-type *M. xanthus* | Tübingen, Germany; [[28, 29](#_ENREF_2)] |
| A66 | Wild-type *M. xanthus* | Tübingen, Germany; [[28, 29](#_ENREF_2)] |
| A88 | Wild-type *M. xanthus* | Tübingen, Germany; [[28, 29](#_ENREF_2)] |
| A96 | Wild-type *M. xanthus* | Tübingen, Germany; [[28, 29](#_ENREF_2)] |
| DK801 | Wild-type *M. xanthus* | Tracy, CA, U.S.A.; Kaiser collection [[29](#_ENREF_3)] |
| DK805 | Wild-type *M. xanthus* | Palo Alto, CA, U.S.A.; Kaiser collection |
| DK816 | Wild-type *M. xanthus* | Ontario, Canada; Kaiser collection [[29](#_ENREF_3)] |
| DK823 | Wild-type *M. xanthus* | Solvang, CA, U.S.A.; Kaiser collection |
| DK836 | Wild-type *M. xanthus* | Albany, NY, U.S.A.; Kaiser collection [[29](#_ENREF_3)] |
| DK852 | Wild-type *M. xanthus* | Saint Louis Zoo, MO, U.S.A.; Kaiser collection |
| DK854 | Wild-type *M. xanthus* | St. Louis, MO, U.S.A.; Kaiser collection |
| DK1622 | Wild-type *M. xanthus* laboratory strain | [[1S](#_ENREF_4)] |
| GVK897 | Wild-type *M. xanthus* | Velicer collection; originally labeled DK897, but genotype differs from report [[2S](#_ENREF_5)] |
| HW-1 | Wild-type *M. fulvus* | Qingdao, Shandong province, China; ATCC |
| Mxx23 | Wild-type *M. xanthus* | Minneapolis, MN, U.S.A.; Reichenbach collection [[29](#_ENREF_3)] |
| Pali | Wild-type *M. xanthus* | Velicer collection |
| DK8601 | A^–^S^–^ *M. xanthus*, *aglB1* Δ*pilA*::tc, Tc^r^ | [[8](#_ENREF_6)] |
| DW1047 | DK8601(P*_pilA_*-SS_OM_-mCherry in pCR2.1), Tc^r^, Km^r^ | [[8](#_ENREF_6)] |
| DW1463 | A^–^S^–^, P*_pilA_*-*traAB* | [[7](#_ENREF_7)] |
| DW1467 | A^–^S^–^, DK8601 Δ*traA* (markerless) | This study |
| DW1468 | DW1467 (pDP23), Km^r^ | This study |
| DW1469 | DW1467 (pDP24), Km^r^ | This study |
| DW1470 | DW1467 (pDP25), Km^r^ | This study |
| DW1471 | DW1467 (pDP26), Km^r^ | This study |
| DW1466 | A^–^S^–^, Δ*tgl*::tc Δ*cglC* (markerless), Tc^r^ | [[7](#_ENREF_7)] |
| DW1472 | DW1466 (pDP23), Tc^r^, Km^r^ | This study |
| DW1473 | DW1466 (pDP24), Tc^r^, Km^r^ | This study |
| DW1474 | DW1466 (pDP25), Tc^r^, Km^r^ | This study |
| DW1475 | DW1466 (pDP26), Tc^r^, Km^r^ | This study |
| DW1476 | DW1467 (pDP27), Tc^r^, Km^r^ | This study |

**Supplemental references**

1S. Wall D, Kolenbrander PE, Kaiser D (1999) The *Myxococcus xanthus* *pilQ* (*sglA*) gene encodes a secretin homolog required for type IV pilus biogenesis, social motility, and development. J Bacteriol 181: 24-33.

2S. Cortina NS, Revermann O, Krug D, Muller R (2011) Identification and characterization of the althiomycin biosynthetic gene cluster in *Myxococcus xanthus* DK897. Chembiochem 12: 1411-1416.
