# Supplementary material for: Molecular Recognition by a Polymorphic Cell Surface Receptor Governs Cooperative Behaviors in Bacteria
Source: PLoS Genet. 2013 Nov 7;9(11):e1003891. doi: 10.1371/journal.pgen.1003891 (PMC3820747; doi:10.1371/journal.pgen.1003891)
Supplement: Table S2 — Primers used in this study. (DOCX) [file pgen.1003891.s005.docx]

Table S2. Primers used in this study.

| **Primer name** | **Purpose** | **Sequence** (5′–3′)* |
| --- | --- | --- |
| PA14-pMAL-*Eco*RI-F | Fusion | CACACAGAATTCGGCGAAAAGGAACCGGAGCT |
| PA14-pMAL-*Pst*I-R | Fusion | CACACACTGCAGTCATCCGCCCTCCGTGTGGAA |
| Δ*traA*-Upstr-*Hind*III-F | Deletion | CACACAAAGCTTGGGTGCTCTTCGAGGTGAT |
| Δ*traA*-Upstr-*Eco*RI-*Pst*I-R | Deletion | CACACAGAATTCATCCTGCAGTGGTGAAGGAGCTACGGGA |
| Δ*traA*-Dwnstr-*Pst*I-F | Deletion | CACACACTGCAGGCGAAGACGATGGAGTGC |
| Δ*traA*-Dwnstr-*Eco*RI-R | Deletion | CACACAGAATTCAGCTCACGTTGAGCGTCAC |
| Δ*traA*-Verification-F | Deletion | GTGGGCGCTGTACCTCCT |
| Δ*traA*-Verification-R | Deletion | GCTCATCGGGTTGCTTCTG |
| P*_pilA_*-*Eco*RI-F | Expression | GACGACGAATTCCGTCATGTTGGACGAGGT |
| P*_pilA_*-ΔSD-*Xba*I-R | Expression | CAGCAGTCTAGAGCCCGCGGATGGGATTAG |
| TraA-RBS-*Xba*I-F | Expression | GACGACTCTAGA*GGAAACCAAGAATAGAAATAGAAAGGAGAATTA***GTG**GGAGATATCCCTCATTG |
| TraA-*Hind*III-R | Expression | GACGACAAGCTTGAAGAGCTGCACGTTGAAG |
| TraA-Env-Full-F | Sequencing | CAACATCTTCGTGCGACATC |
| TraA-Env-Full-R | Sequencing | CCAGGTTGATCTTGCTCAGG |
| DK816-SR1 | Sequencing | GACTCCATGAACGCATTGAA |
| DK816-SR2 | Sequencing | TAGCTTGGCTGCTGAATCGT |
| MX6895-SF2 | Sequencing | TCACTGTCTTGTCGGTGTGC |
| MX6895-SR2 | Sequencing | GTTGATGTCGTCTGGGAAGG |
| MX6895-SF1 | Sequencing | GTGGGAGATATCCCTCATTGTT |
| MX6895-SR1 | Sequencing | TGCGTTGCAGTGGTAGAAAG |
| MX6895-SF3 | Sequencing | ACTTCGTGAACTCCGTTGGT |
| MX6895-SR3 | Sequencing | GTTGAGGTTGGCGCAGTG |
| MX6895-SF4 | Sequencing | CTGCGACTCCTCGCTCTT |
| MX6895-SR4 | Sequencing | CCATTGGAGAGGCAGAAGG |
| MX6895-Gap1 | Sequencing | CCATCGTCAAGCTCAACACA |
| DK823-R2 | Sequencing | AATGTTTCGGTGTCACAAGC |
| DK823-FW2 | Sequencing | GATTTCGCGGTTACCTGAAC |
| DK823-FW3 | Sequencing | TCAACGTCATCGAGGACAAC |
| DK823-R3 | Sequencing | CGTTCTTGCACTCGACACAT |

*Restriction sites are underlined, start codon is in bold and artificial Shine-Dalgarno region is in italics.
